# Supplementary material for: An evolutionary NS1 mutation enhances Zika virus evasion of host interferon induction
Source: Nat Commun. 2018 Jan 29;9:414. doi: 10.1038/s41467-017-02816-2 (PMC5788864; doi:10.1038/s41467-017-02816-2)
Supplement: Supplementary file 2 — Supplementary Information [file 41467_2017_2816_MOESM2_ESM.pdf]

Supplementary Figure 1

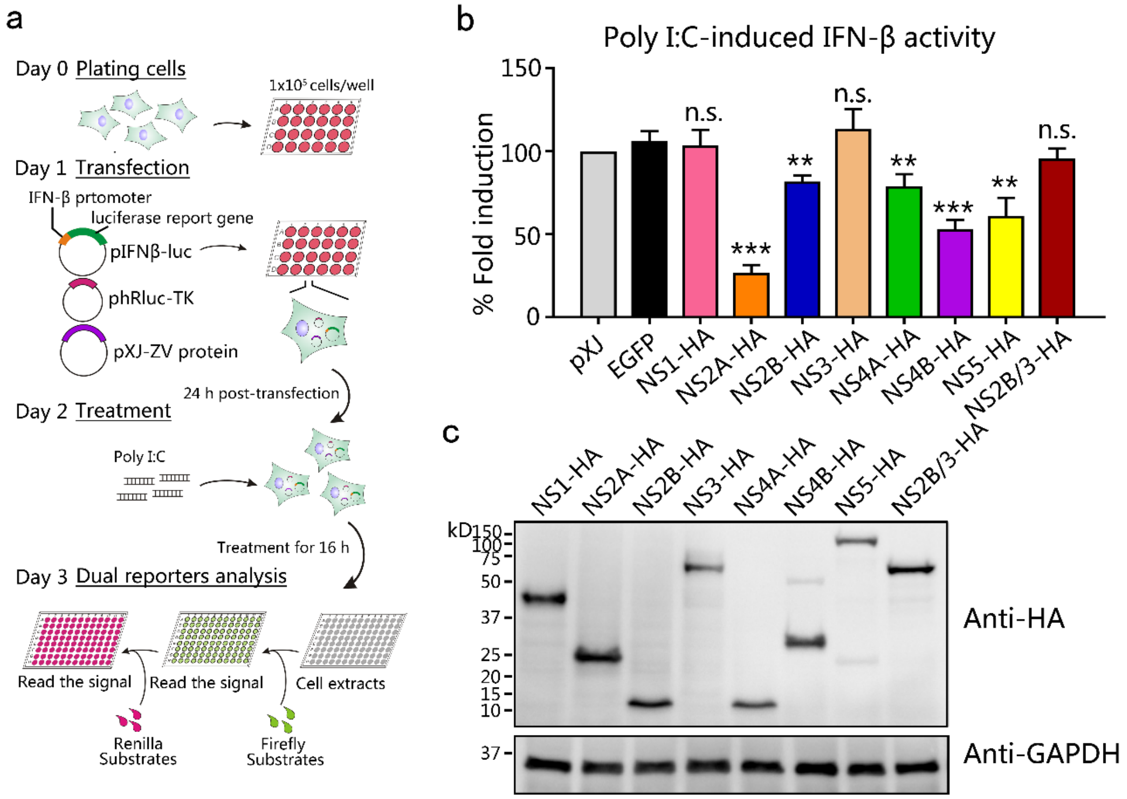

Supplementary Figure 1. Screening of ZIKV proteins that antagonize IFN-β induction.
(a) Luciferase assay scheme. HEK-293T cells were seeded in 24-well plate (1×10^5 cells/well) and transfected with reporter plasmid pIFN-β-luc, phRluc-TK, and expression viral proteins plasmids. At 24 h post-transfection, cells were stimulated with poly I:C for 16 h. Cells were lysed after stimulation and luciferase activity was determined with the Dual-Luciferase Reporter Assay System. The data were determined by normalization of the firefly luciferase activities to the renilla luciferase activities. (b) IFN-β promoter luciferase activities in HEK-293T cells co-transfected with ZIKV individual proteins expressing plasmids, EGFP encoding plasmid or empty vector as controls. After 24 h post-transfection, cells were treated with 2 µg poly I:C for 16 h. Data were normalized first by renilla luciferase values, and then normalized by none stimulated samples to obtain fold induction. Empty vector samples were set to 100% to get percentage of fold induction. Data are from three independent experiments, each one in triplicate (mean ± SD of fold change). Statistics were determined by unpaired Student's t test, $^{*}P < 0.05$, $^{**}P < 0.01$, $^{***}P < 0.001$, or no significance (n.s.). (c) Protein expression level were determined by using lysates from transfected samples are shown as controls, and the original uncropped blot can be found in Supplementary Fig. 8.

Supplementary Figure 2

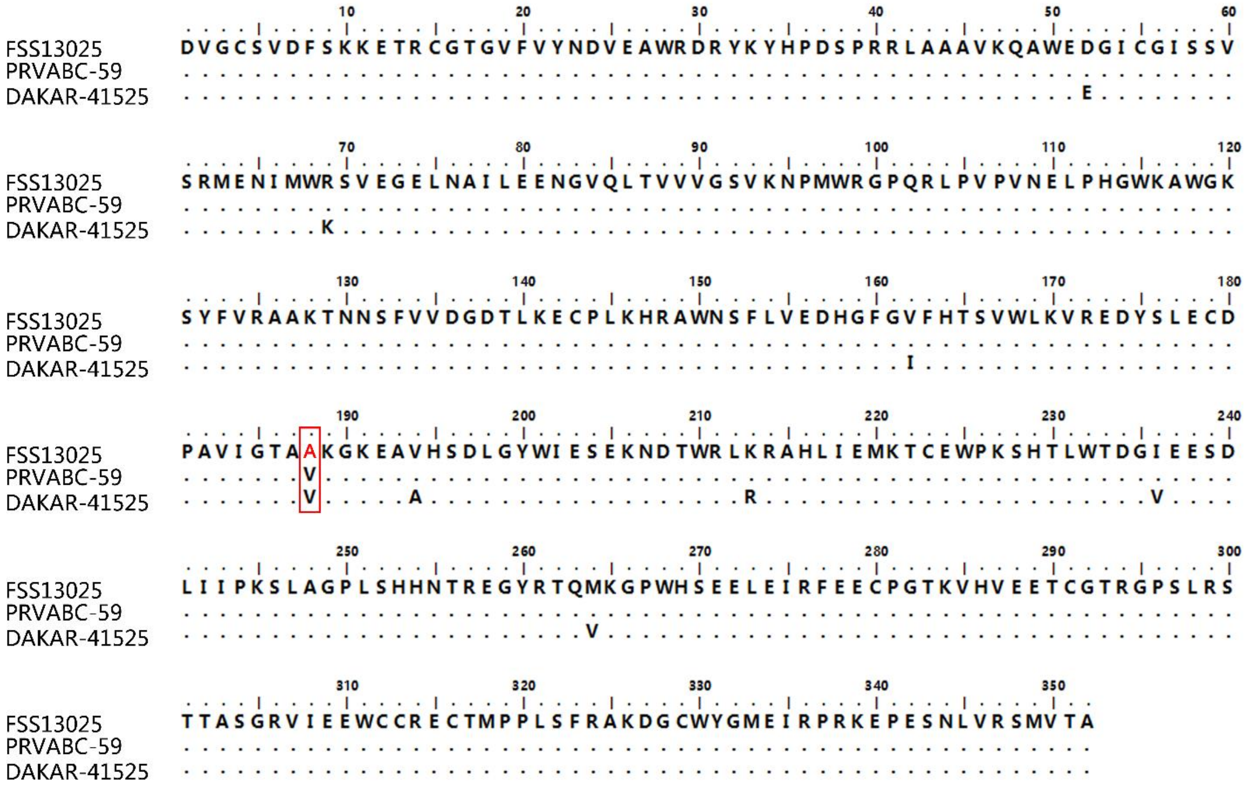

Supplementary Figure 2. Sequence analyses.
Amino acids sequence alignment of NS1 proteins from FSS13025 (Asian lineage, GenBank number KU955593.1), PRVABC-59 (American lineage, GenBank number KU501215), and Dakar-41525 (African lineage, GenBank number KU955591.1). Residue 188 was highlighted in red.

Supplementary Figure 3

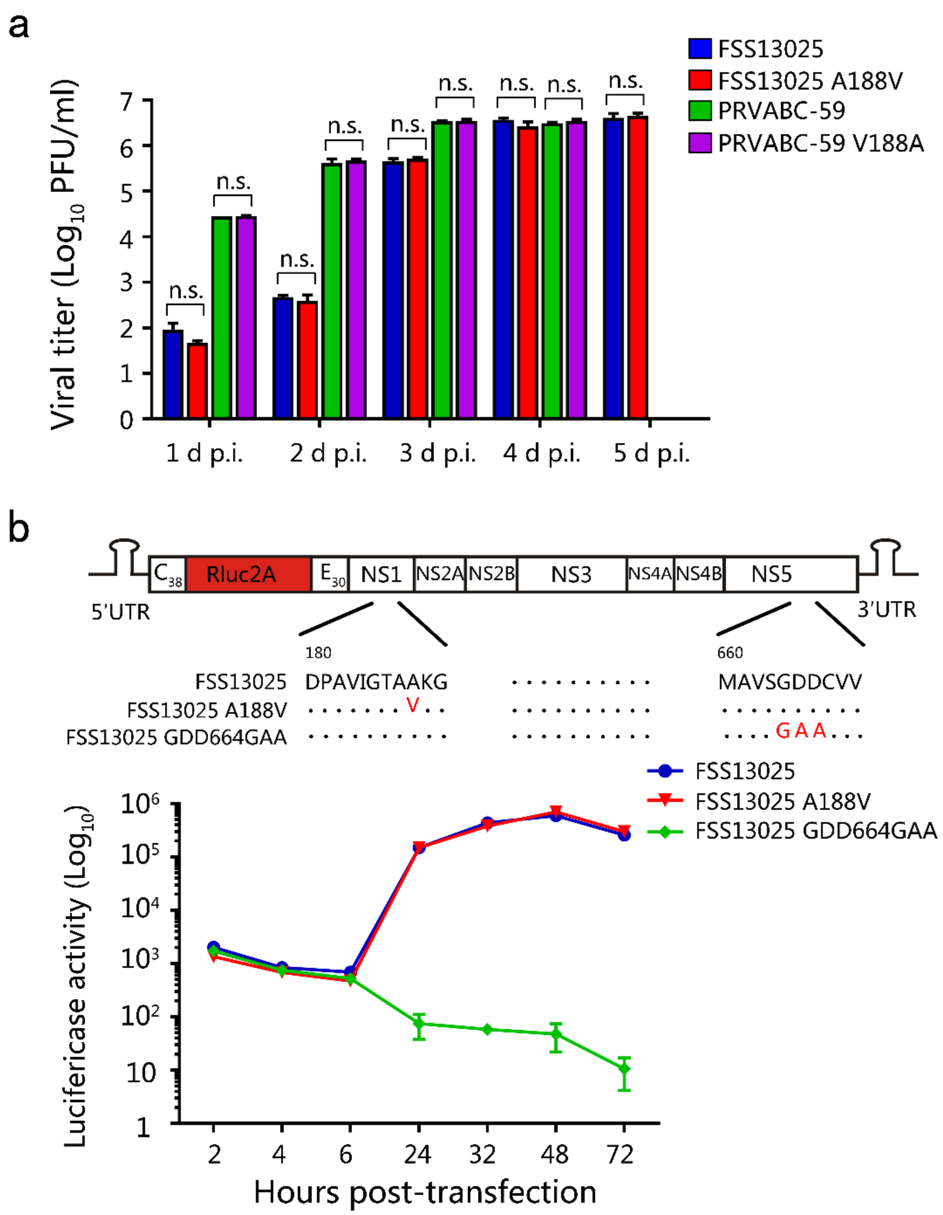

Supplementary Figure 3. The identity of amino acid 188 of NS1 modulates IFN-β production during ZIKV infection of human cell lines.
(a) Comparison of growth kinetics of FSS13025 WT, FSS13025/A188V, PRVABC-59 WT and PRVABC-59/V188A. Vero cells were seeded into 6-well-plate ($8×10^5$ cells/well). At 16 h post-seeding, cells were infected with viruses at an MOI of 0.01. After 1 h incubation at 37 °C, supernatant was removed and cells were washed twice with PBS, then 3 ml fresh 2% medium was added to each well. Supernatant was collected according to the time points and centrifuged at 500 g for 5 min prior to storage at -80°C. Viral titer was determined by plaque assay as described above. (b) Replicon transient

transfection assay. Upper panel shows the scheme of ZIKV WT and mutants replicons with renilla luciferase reporter. Bottom panel shows luciferase activity of ZIKV replicons. Replicon RNAs were *in vitro* transcribed and electroporated into Vero cells. Cells were seeded into 12-well plate (3.2×10^5 per well) after electroporation and incubating at 37 °C. At various time points, cells were washed twice with PBS and lysed in 200 µl lysis buffer. Luciferase signals were measured through mixing with renilla luciferase substrates (Promega) and read by Cytation 5 (Biotek) according to the manufacturer's instructions. Data are mean ± SD from three independent replicates. Statistical values were analyzed by unpaired Student’s t test, no significance (n.s.).

Supplementary Figure 4

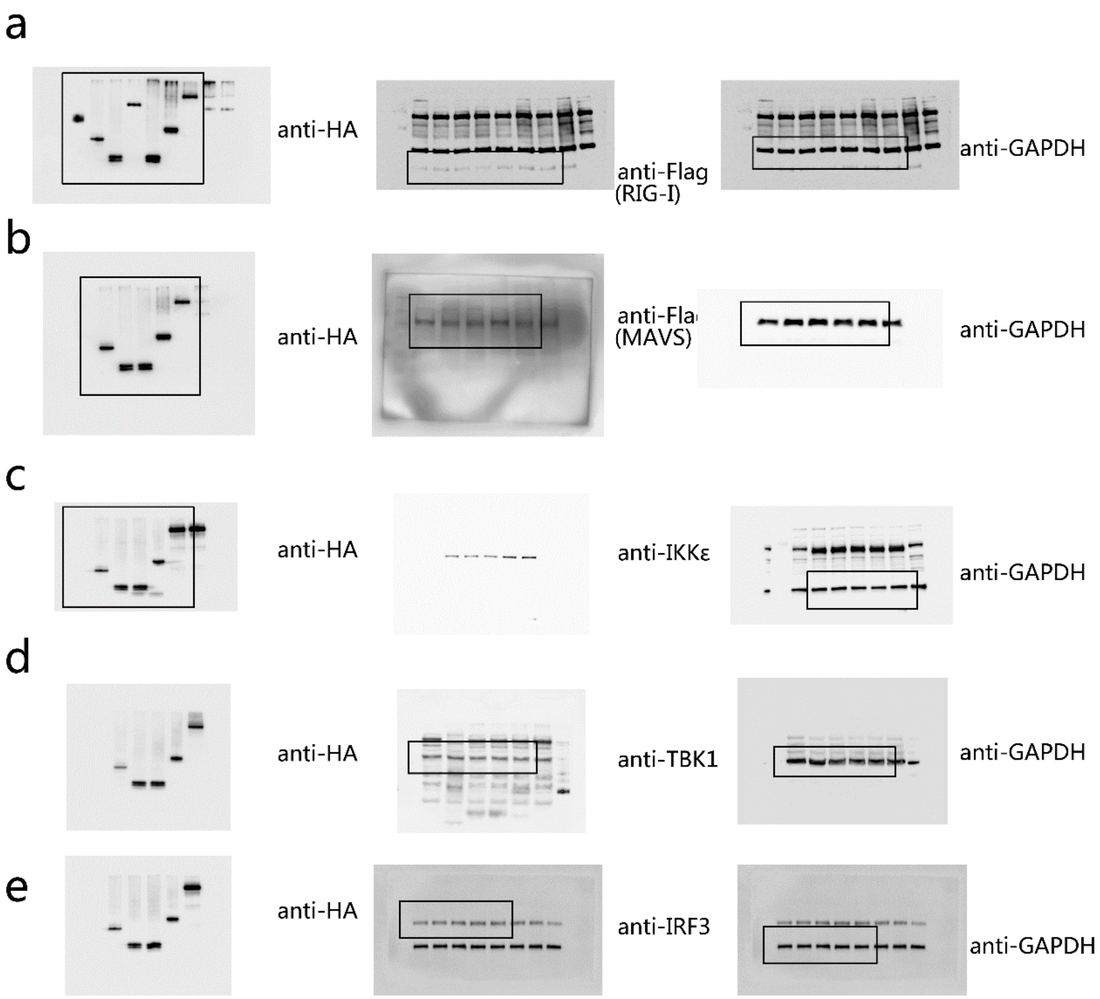

Supplementary Figure 4. Uncropped images of western blots.
(a) For Fig. 1b. (b) For Fig. 1c. (c) For Fig. 1d. (d) For Fig. 1e. (e) For Fig. 1f.

Supplementary Figure 5

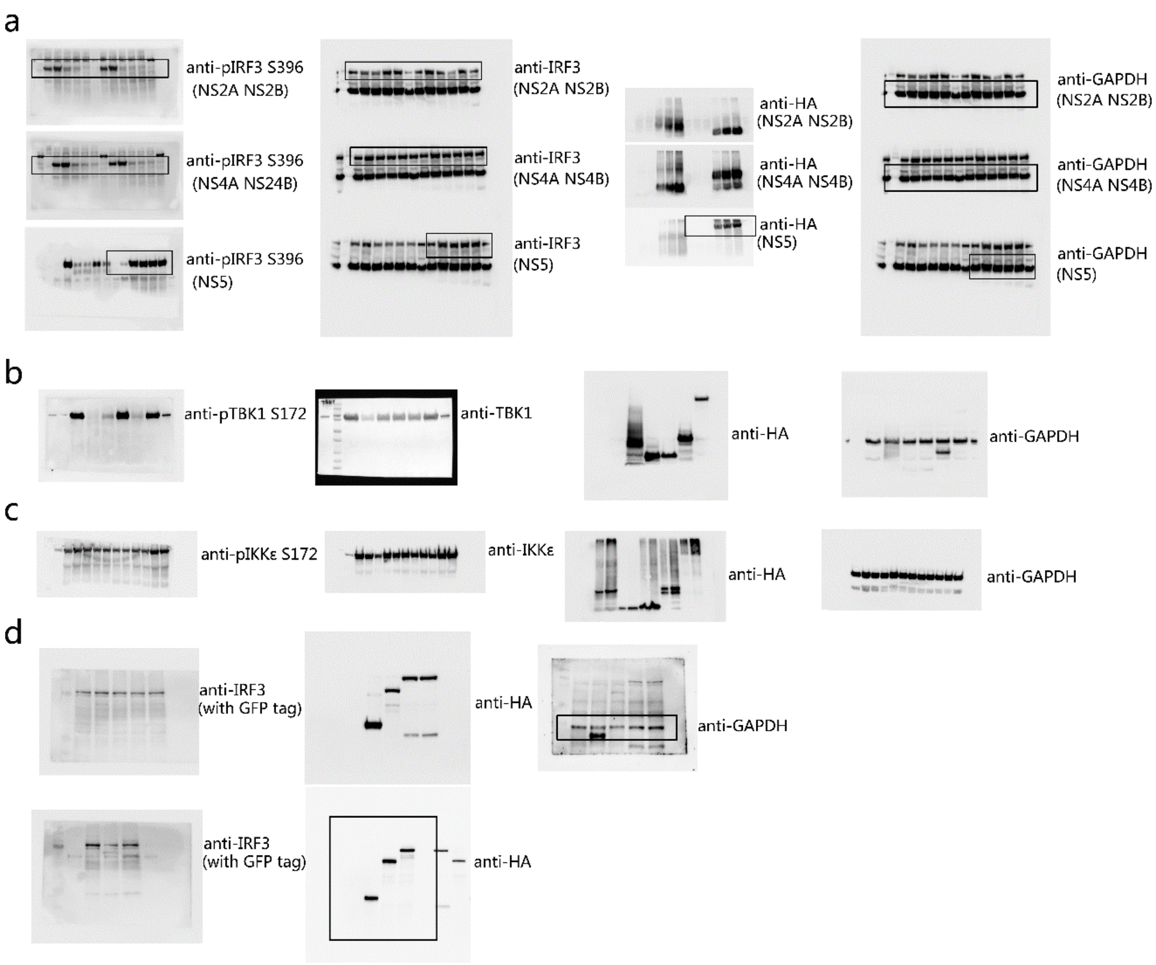

Supplementary Figure 5. Uncropped images of western blots.
(a) For Fig. 2a. (b) For Fig. 2b. (c) For Fig. 2c. (d) For Fig. 2d.

Supplementary Figure 6

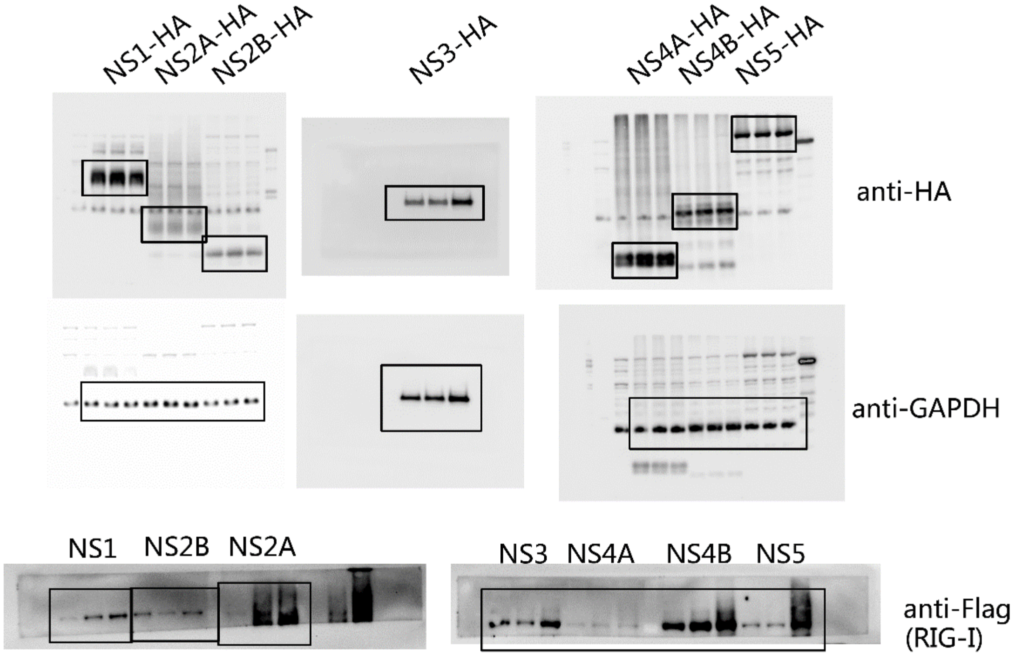

Supplementary Figure 6. Uncropped images of western blots.
For Fig. 3a.

Supplementary Figure 7

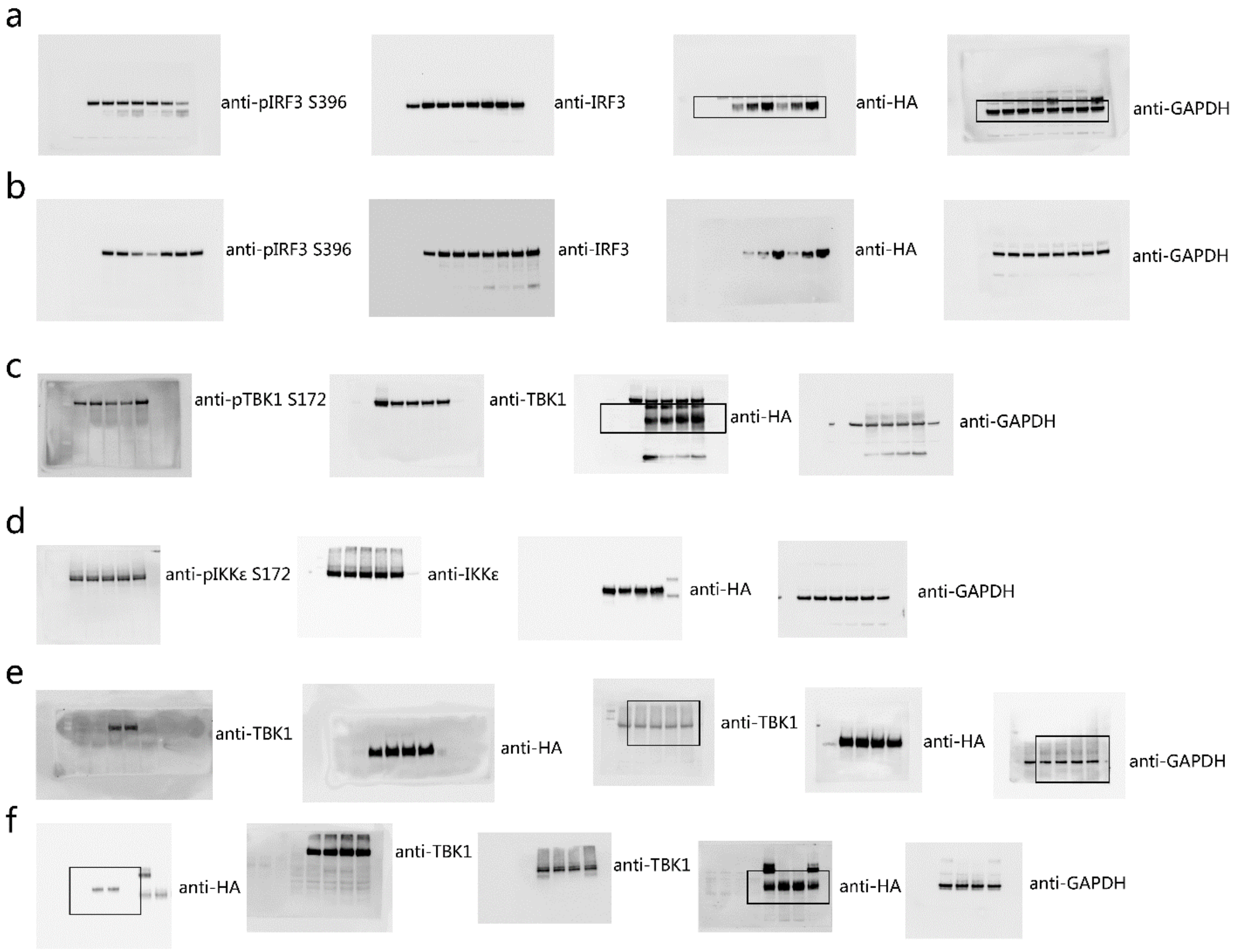

Supplementary Figure 7. Uncropped images of western blots.
(a) For Fig. 4a. (b) For Fig. 4b. (c) For Fig. 4c. (d) For Fig. 4d. (e) For Fig. 4e. (f) For Fig. 4f.

Supplementary Figure 8

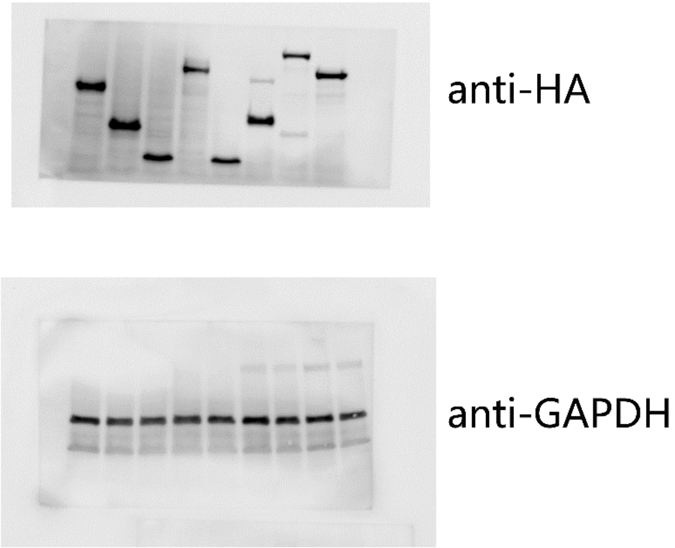

Supplementary Figure 8. Uncropped images of western blots.
For Supplementary Fig. 1c.

Supplementary Table 1. Amino acid differences between ZIKV FSS13025 and PRVABC-59 strains[a]

Amino Acid Position	FSS13025	PRVABC-59	Location
80	I	T	C
106	T	A	C
123	V	A	prM
130	N	S	prM
139	S	N	prM
151	M	L	prM
763	V	M	E
982	A	V	NS1
1274	P	L	NS2A
1477	A	T	NS2B
2086	Y	H	NS3
2611	A	V	NS5
2634	M	V	NS5
3392	M	V	NS5
3403	V	M	NS5

[a]Different amino acids are indicated by their residue positions of specific viral genes. The GenBank numbers for strains FSS13025 and PRVABC-59 are KU955593.1 and KU501215, respectively.

Supplementary Table 2. Sequences of PCR primers used in this study

Primer name	Sequence (5′–3′)[a]
E24-NS1-HA-F	GATGCGGCCGCACCATGAATGGATCTATTTCCCTTATGTGCTTG
E24-NS1-HA-R	GGAACATCGTATGGGTAGGATCCTGCAGTCACCATTGACCTTACTAA
SPG-C16-NS2A-HA-F1	GATGCGGCCGCACCATGGGAGTCAAAGTTCTGTTTGCC
SPG-C16-NS2A-HA-R1	CTTTCTGGTTCTTTCCTGGGAGTCACGGCCTCGGCCACAGCG
SPG-C16-NS2A-HA-F2	GCCGTGACTCCCAGGAAAGAACCAGAAAGT
SPG-C16-NS2A-HA-R2	GGAACATCGTATGGGTAGGATCCCCGCTTCCCACTCCTTGTGAGCAAC
NS2B-HA-F	GATGCGGCCGCACCATGAGCTGGCCCCCTAGTGAAGTACT
NS2B-HA-R	CTGGAACATCGTATGGGTAGGATCCCCTTTTTCCAGTCTTCACATACAC
NS3-HA-F	GATGCGGCCGCACCATGAGTGGTGCTCTATGGGATGTGCCT
NS3-HA-R	GGAACATCGTATGGGTAGGATCCTCTTTTCCCAGCGGCAAACTCTTTGA
NS4A-HA-F	GATGCGGCCGCACCATGGGAGCGGCTTTTGGAGTGATGG
NS4A-HA-R	GGAACATCGTATGGGTAGGATCCTCTTTGCTTTTCTGGCTCAGGTATG
2K-NS4B-HA-F	GATGCGGCCGCACCATGTCTCCCCAGGACAACCAAATGGCA
2K-NS4B-HA-R	GGAACATCGTATGGGTAGGATCCACGTCTCTTGACCAAGCCAGCGT
NS5-HA-F	GATGCGGCCGCACCATGGGAGGTGGAACGGGAGAGACCCTGGGA
NS5-HA-R	GGAACATCGTATGGGTAGGATCCTAGCACTCCAGGTGTGGACCCTT
NS5-MTase-R	GGAACATCGTATGGGTAGGATCCCCGCGTGCCAGAGCCGAGATTCAC
NS5-RdRp-F	GTGCGGCCGCACCATGAAGATCATTGGTAACCGCATTG
HA-C ter-R	GACCTCGAGCTAAGCGTAATCTGGAACATCGTATGGGTAGGATCC
FSS-NS1-A188V-F	CCGTCATTGGAACAGCCGTTAAGGGAAAGGAGGCTG
FSS-NS1-A188V-R	GCCTCCTTTCCCTTAACGGCTGTTCCAATGACGGCTGG
PRV-NS1-V188A-F	GTTATTGGAACAGCTGCTAAGGGAAAGGAGGCTGTAC
PRV-NS1-V188A-R	CCTCCTTTCCCTTAGCAGCTGTTCCAATAACGGC

[a] Restriction enzyme site sequences are underlined.

Supplementary Table 3. Sequences of qRT-PCR primers and probe.

**Primer name**	**Sequence (5′–3′)**
H_IFNβ-F	CATTACCTGAAGGCCAAGGA
H_IFNβ-R	CAATTGTCCAGTCCCAGAGG
H_GAPDH-F	TGTTGCCATCAATGACCCCTT
H_GAPDH-R	CTCCACGACGTACTCAGCG
ZIKV-1193F	CCGCTGCCCAACACAAG
ZIKV-1269R	CCACTAACGTTCTTTTGCAGACAT
M_IFNβ-F	ACCTACAGGGCGGACTTCAAG
M_IFNβ-R	GATGGCAAAGGCAGTGTAACTCTT
M_GAPDH-F	AGGTCGGTGTGAACGGATTTG
M_GAPDH-R	TGTAGACCATGTAGTTGAGGTCA
ZIKV-probe	FAM/AGCCTACCT/ZEN/TGACAAGCAATCAGACACTCAA/3IABkFQ
